# A Case of Vigabatrin Toxicity Mimicking Bilateral Thalamic Infarcts on MRI

**DOI:** 10.7759/cureus.45049

**Published:** 2023-09-11

**Authors:** Trevor J Lockard, Geetanjali Rathore

**Affiliations:** 1 Pediatric Neurology, University of Nebraska Medical Center, Omaha, USA; 2 Pediatric Neurology, Children's Hospital & Medical Center, Omaha, USA

**Keywords:** vigabatrin, mri, infantile spasms, tuberous sclerosis, diffusion restriction, thalamus, globus pallidus, drug reaction

## Abstract

A 20-month-old female with a past medical history of tuberous sclerosis, epilepsy, and infantile spasms treated with vigabatrin presented for surveillance MRI for multiple brain hamartomatous lesions and subependymal nodules. MRI showed new restricted diffusion to bilateral thalami and globi pallidi. This finding was concerning for bilateral thalamic strokes, with differential to include infection, metabolic etiologies, or toxic injuries. Without focal or diffuse neurologic symptoms or additional MRI lesions to suggest an acute or chronic pathology, it was determined the MRI signal changes were likely induced by vigabatrin. Vigabatrin therapy was continued, and a repeat MRI 17 months later showed a resolution of the diffusion restriction with no residual sequelae. Vigabatrin-induced MRI abnormalities are an uncommon adverse effect of therapy for infantile spasms, with adverse events being most common in young infants. It is crucial to consider this adverse drug effect in an asymptomatic patient presenting with these MRI lesions as the findings are otherwise suggestive of a serious disease process, such as an inborn error of metabolism, requiring expensive and invasive workup.

## Introduction

Vigabatrin is one of three drugs, the others being adrenocorticotropic hormone (ACTH) and prednisone, considered to be the gold standard in the treatment of infantile spasms, a type of childhood epilepsy characterized by clusters of seizures with rhythmic flexion and extension of the head and trunk [1]. Patients with tuberous sclerosis have a high probability of developing infantile spasms, which may even be the presenting symptom of their illness [1]. Prompt treatment is critical to ensure continued development and prevent intellectual disability; however, each of the three drugs comes with risks of adverse events [2]. Vigabatrin is known to cause unusual MRI changes in young patients; however, it is less reported to occur in tuberous sclerosis patients [3]. We present a case of a tuberous sclerosis patient with infantile spasms who developed MRI changes secondary to vigabatrin use, and we discuss the significance of these findings for pediatric providers seeking to optimize care.

This article has been presented previously as a poster at the Child Health Research Initiative Annual Conference in Omaha, Nebraska on May 11, 2023.

## Case presentation

**Figure 1:**
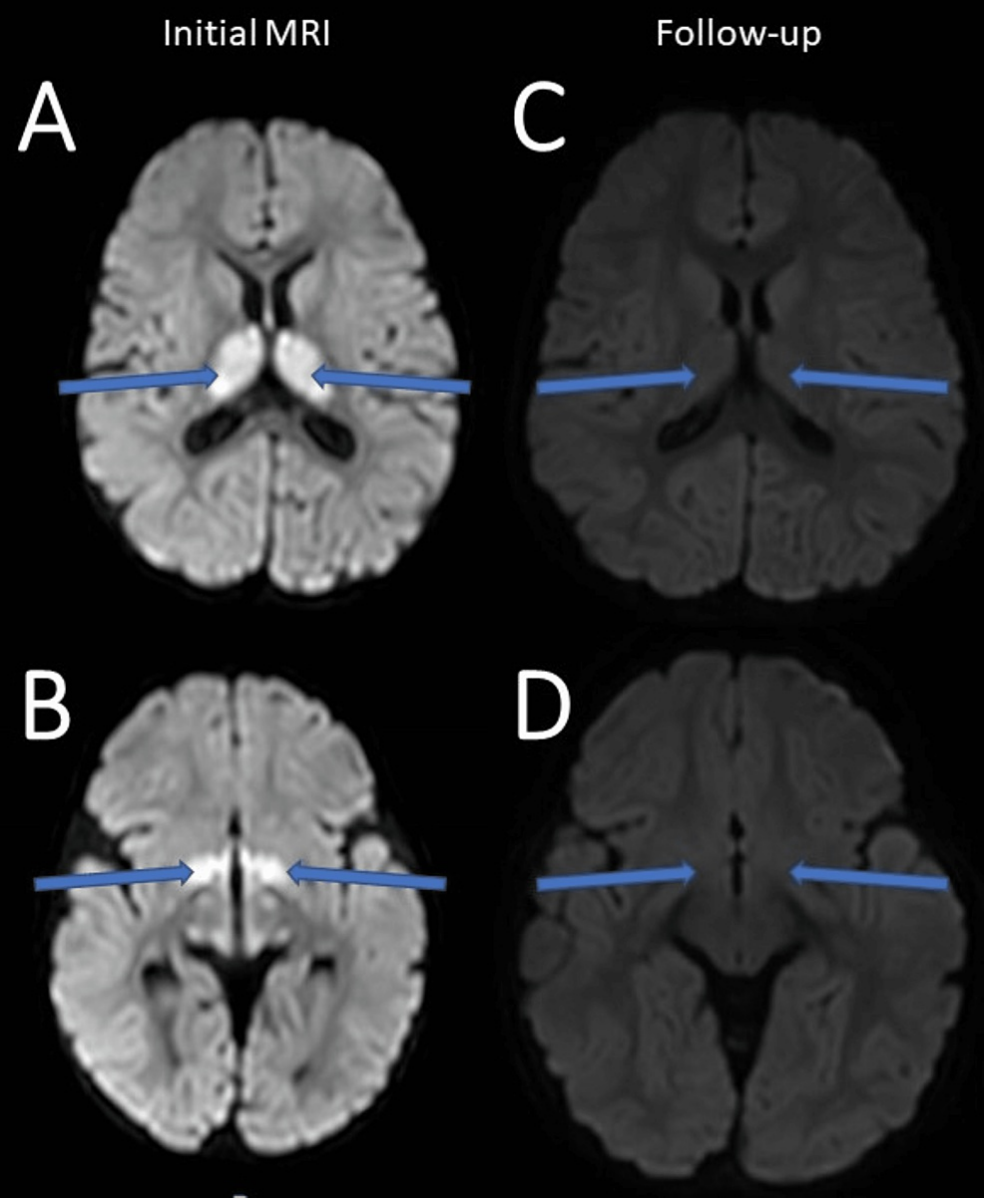
Vigabatrin-induced transient hyperintensities with diffusion restriction in bilateral (A) thalami and (B) globi pallidi on diffusion-weighted MRI. Follow-up MRI in 17 months showed total resolution of both hyperintensities (C-D).

A 20-month-old female with a history of tuberous sclerosis and refractory infantile spasms presented for a follow-up of her annual surveillance brain MRI. She had been seizure-free for four months on the current regimen of vigabatrin 150 mg/kg/day and was being weaned off ACTH injections. Her MRI of the brain unexpectedly showed new restricted diffusion to bilateral thalami and globi pallidi (Figures 1A, 1B). This finding was concerning for bilateral thalamic strokes; however, due to the patient being asymptomatic with a normal exam, the differential was expanded to infection, metabolic etiologies, or toxic injuries. Entities to be considered included viral or bacterial encephalitis, toxoplasmosis, West syndrome, leukodystrophies, Wilson disease, hypoxic-ischemic encephalopathy, methanol ingestion, or cyanide poisoning. Of note, isolated thalamic MRI signal changes are typically due to focal (ischemic, malignant, infectious) causes versus systemic ones [4]. Differentiating these entities relies on clinical presentation, such as lethargy and fever in infections, other organ involvement in metabolic syndromes, or respiratory distress and altered mental status characteristic of many toxic ingestions. Without focal or diffuse neurologic symptoms or additional MRI lesions to suggest an acute or chronic pathology, it was determined the MRI signal changes were likely induced by vigabatrin. Further studies were not obtained, and the patient was followed closely as an outpatient. Vigabatrin therapy was continued, and a repeat MRI 17 months later showed resolution of the diffusion restriction with no residual sequelae (Figures 1C, 1D). The patient never developed extrapyramidal symptoms to correlate with her MRI findings during this time, and she remains on vigabatrin therapy without adverse effects.

## Discussion

Vigabatrin is an irreversible gamma-aminobutyric acid transaminase (GABA-T) inhibitor that has an antiseizure effect by increasing levels of gamma-aminobutyric acid (GABA) in the brain. This drug is first-line therapy for infantile spasms in children under two years of age and is indicated for use in refractory complex partial seizures in children over two years [5]. Unique adverse effects of this antiseizure medication include irreversible vision loss and unexplained asymptomatic MRI changes. These MRI changes are generally not seen in patients older than two, making this 20-month-old one of the oldest children reported in the literature to have this reaction. The increased T2-weighted MRI signal in infants with infantile spasms treated with vigabatrin appears to be transient and dose-dependent and may resolve even with continued vigabatrin therapy [3,6]. While changes are commonly reported in the thalami and globi pallidi, one case series demonstrated similar MRI changes, which were found not only in the aforementioned locations but also in the dentate nuclei, medial longitudinal fasciculus, and medulla [3]. The authors believed these changes resulted either from transient Na-K-ATPase pump dysfunction or neuronal vacuolation, although there is not yet consensus on the pathophysiology of this adverse reaction [3]. None of the seven patients with tuberous sclerosis included in this case series had MRI changes [3]. More recently, however, a case of a patient with MRI changes and tuberous sclerosis, as well as rare cases of reversible extrapyramidal symptoms correlated with vigabatrin-induced MRI changes, have been described [6,7].

## Conclusions

Vigabatrin, while generally safe and effective, may cause unexpected abnormalities on MRI. Maintaining a high suspicion for this adverse reaction in patients under two years old is critical, as the differential for such abnormalities can be broad. Furthermore, in patients with underlying systemic illnesses like tuberous sclerosis, developmental delays and disabilities are common and may confound evaluation. In the rare case that vigabatrin-induced MRI abnormalities cause extrapyramidal symptoms, vigabatrin should be permanently stopped. If the patient is asymptomatic, he or she may continue to take vigabatrin as prescribed, and the MRI abnormalities should self-resolve. Further expensive and invasive workup is unnecessary and should be avoided.

## References

[1] Pavone P, Polizzi A, Marino SD, Corsello G, Falsaperla R, Marino S, Ruggieri M (2020). West syndrome: a comprehensive review. Neurol Sci.

[2] Ramantani G, Bölsterli BK, Alber M (2022). Treatment of infantile spasm syndrome: update from the interdisciplinary guideline committee coordinated by the German-speaking Society of Neuropediatrics. Neuropediatrics.

[3] Pearl PL, Vezina LG, Saneto RP (2009). Cerebral MRI abnormalities associated with vigabatrin therapy. Epilepsia.

[4] Hegde AN, Mohan S, Lath N, Lim CC (2011). Differential diagnosis for bilateral abnormalities of the basal ganglia and thalamus. Radiographics.

[5] (2022). Vigabatrin: drug information. https://www.uptodate.com/contents/vigabatrin-drug-information?search=vigabatrin&source=panel_search_result&selectedTitle=1~25&usage_type=panel&kp_tab=drug_general&display_rank=1.

[6] Craft JF, Cardenas AM (2021). Vigabatrin-associated reversible MRI abnormalities in an infant with tuberous sclerosis. J Radiol Case Rep.

[7] Dill P, Datta AN, Weber P, Schneider J (2013). Are vigabatrin induced T2 hyperintensities in cranial MRI associated with acute encephalopathy and extrapyramidal symptoms?. Eur J Paediatr Neurol.

